# Bacterial Attachment and Biofilm Formation on Antimicrobial Sealants and Stainless Steel Surfaces

**DOI:** 10.3390/foods11193096

**Published:** 2022-10-05

**Authors:** Luminita Ciolacu, Elena Zand, Carmen Negrau, Henry Jaeger

**Affiliations:** Institute of Food Technology, University of Natural Resources and Life Sciences (BOKU), 1190 Vienna, Austria

**Keywords:** biofilm, gram-positive bacteria, antimicrobial agent, food hygiene, sealant, stainless steel

## Abstract

Biofilms are highly resistant to external forces, especially chemicals. Hence, alternative control strategies, like antimicrobial substances, are forced. Antimicrobial surfaces can inhibit and reduce microbial adhesion to surfaces, preventing biofilm formation. Thus, this research aimed to investigate the bacterial attachment and biofilm formation on different sealants and stainless steel (SS) surfaces with or without antimicrobials on two Gram-positive biofilm forming bacterial strains. Antimicrobial surfaces were either incorporated or coated with anti-microbial, -fungal or/and bactericidal agents. Attachment (after 3 h) and early-stage biofilm formation (after 48 h) of *Staphylococcus capitis* (*S. capitis*) and *Microbacterium lacticum* (*M. lacticum*) onto different surfaces were assessed using the plate count method. In general, bacterial adhesion on sealants was lower compared to adhesion on SS, for surfaces with and without antimicrobials. Antimicrobial coatings on SS surfaces played a role in reducing early-stage biofilm formation for *S. capitis*, however, no effects were observed for *M. lacticum*. *S. capitis* adhesion and biofilm formation were reduced by 8% and 25%, respectively, on SS coated with an antimicrobial substance (SS_4_M), compared to the same surface without the antimicrobial coating (SS_4_control). Incorporation of both antifungicidal and bactericidal agents (S_5_FB) significantly reduced (*p* ≤ 0.05) early-stage biofilm formation of *M. lacticum,* compared to the other sealants incoportating either solely antifungal agents (S_2_F) or no active compound (S_control). Furthermore, the thickness of the coating layer correlated weakly with the antimicrobial effect. Hence, equipment manufacturers and food producers should carefully select antimicrobial surfaces as their effects on bacterial adhesion and early-stage biofilm formation depend on the active agent and bacterial species.

## 1. Introduction

Biofilms in the food industry are a primary cause for cross-contamination and metal corrosion [1,2]. Spoilage and pathogenic bacteria can colonize a wide range of surfaces commonly found in the food industry, like rubber, polypropylene, plastic, glass, or stainless steel (SS) [3]. SS is widely used because of its high corrosion resistance and superior mechanical properties [4], while polymer materials, such as polyethylene (PE) and polyvinyl chloride (PVC), are cost-effective options known to inhibit biofilm growth and corrosion [5].

Nowadays, cleaning and disinfectant agents are typically used to reduce or eliminate bacterial biofilms [6,7]. However, biofilms show increased resistance against chemicals compared to planktonic cells [8]. Hence, alternative methods to control biofilm formation, like modification of surfaces to prevent bacterial adhesion, are needed [2,6]. Different techniques can be applied to develop antimicrobial surfaces: (i) incorporation of antimicrobial agents into the surface material; (ii) deposition of antimicrobial coatings on the surface (=surface coating), or (iii) changes in relevant surface characteristics (e.g., roughness, surface free energy, hydrophobicity) [9,10]. Furthermore, in the context of increased environmental awareness, antimicrobial surfaces have the potential to become sustainable, eco-friendly alternatives replacing chemical cleaning and disinfection agents [11,12].

Moreover, antimicrobial surfaces can be divided further into (i) antimicrobial (bactericidal or/and fungicidal) and (ii) antibiofouling surfaces. The antimicrobial effect is achieved by releasing biocides or by contact killing which is specifically targeting i.e., the cell membrane of bacteria for bactericidal substances [13,14]. In contrast, antibiofouling surfaces are created to resist microbial accumulation and adsorption, thus limiting the attachment of the microorganisms on the surface and the biofilm formation [14,15]. In the present study antimicrobial (bactericidal, fungicidal or a combined approach) surfaces are considered. More details on antibiofouling surfaces can be found in the following review [16].

In recent years, significant progress has been made, and many different types of antimicrobial surfaces proved effective against surface-associated biofilms [17,18]. The mode of action was mainly related to the smaller contact area between bacteria and coating or to the hydrophilic properties of coatings [19,20,21]. However, the variation in results is still high due to the type of antimicrobial agent used, mode of applying the coatings, biofilm assessment method, or bacterial strains tested [22,23,24].

The aim of this study was to assess the attachment and early-stage biofilm formation of two Gram-positive bacterial strains in a static environment on different sealants and SS surfaces, some of them incorporating or coated with active antimicrobial, -fungal and/or bactericidal agents. Hence, this study provides valuable insights for equipment manufacturers and food processors regarding the efficacy of different surfaces in reducing biofilm formation.

## 2. Material and Methods

### 2.1. Bacterial Strains and Culture Conditions

Two Gram-positive bacteria frequently found in the food industry, especially in the milk or meat industry were selected because of their strong biofilm forming ability [25]. *Microbacterium lacticum* (*M. lacticum*) D84 (EF 204392) was isolated from extended shelf-life (ESL) milk, and *Staphylococcus capitis* subsp. *capitis* (*S. capitis)* was isolated from an air decontamination step prior to packaging at a meat production facility. Bacterial stock and working cultures were maintained and prepared according to *Zand* et al. [25]. The isolates were preserved in a 50% (*v*/*v*) glycerol stock at −80 °C. To obtain a stock culture, bacteria were sub-cultured overnight in tryptic soy broth (TSB; Carl Roth, Karlsruhe, Germany) at 37 °C and in 0.8% (*v*/*v*) skimmed milk broth (MB; Carl Roth) at 30 °C, for *S. capitis* and *M. lacticum*, respectively. Subsequently, stock cultures were then streaked onto either tryptic soy agar (TSA) or milk agar (MA) (Carl Roth) and incubated at 37 °C or 30 °C overnight and stored at 4 °C. Before each experiment, one colony was inoculated in 10 mL fresh TSB or MB, and the optical density_600_ (OD) was standardized to 0.1 to obtain a working culture.

### 2.2. Surface Types

Six sealants and five SS surface coupons (4 cm^2^) were selected, considering surfaces with and without antibactericidal, -fungal, or -microbial agents according to the manufacturer’s data (Table 1), to test their efficiency against biofilm-forming bacteria.

A surface with 1K silyl-modified polyethers (MS) polymer-basis without fungicidal or bactericidal active ingredients, referred to as hybrid glue 007 (S_control, EVT Dichtstoffe GmbH, Korntal-Münchingen, Germany), was included as a control. Additionally, one-component silicon-based sealants incorporating active fungicidal agents: Joint HPA (S_1_F), clean room sanitary HPCR (S_2_F) and sanitary HPS (S_3_F) (all EVT Dichtstoffe GmbH, Korntal-Münchingen, Germany), were tested. Other sealant materials with silane-terminated hybrid-polymer (STP) or acetat base, one incorporating active antimicrobial agents, referred to as Novasil (S_4_M, Hermann Otto GmbH, Fridolfing, Germany) and one incorporating both fungicidal and bactericidal active ingredients named 450 sanitary (S_5_FB, Ramsauer GmbH & Co KG, Upper Austria, Austria), respectively, were included. All SS surfaces (Brucha GmbH, Michelhausen, Austria) were SS type AISI-304 and differ in respect to the type and size of the coating applied on the metal: Polyester foil with 25 µm thickness (SS_1), polyurethan-polyamide foil with 50 µm thickness (SS_2), Niro V2A standard alloy (SS_3) as well as PVC foil with 150 µm thickness (SS_4_control, control surface) and PVC foil with 150 µm thickness including an antimicrobial coating (SS_4_M).

All coupons were cleaned before use, according to Zand, et al. [25], with minor modifications. The coupons were soaked in acetone (Roth, Karlsruhe, Germany) for 30 s, rinsed with distilled water, and soaked again in 1N NaOH (Roth, Karlsruhe, Germany) for 1 h. A final rinse with distilled water was carried out prior to sterilization (121 °C for 15 min).

### 2.3. Bacterial Adhesion and Biofilm Formation

Bacterial adhesion and biofilm formation assays were performed as described by Zand, et al. [25]. Briefly, coupons were immersed in a bacterial culture at a level of ~2 log CFU cm^−2^ (~7.5 CFU mL^−1^) in a small petri dish (35 × 10 mm; Greiner Bio-One, Kremsmünster, Austria) and incubated at 30 °C for *M. lacticum* and at 37 °C for *S. capitis*. To assess adhesion, bacterial cells were enumerated after 3 h of incubation. Early-stage biofilm formation was examined after 48 h. A washing step was performed, after the first 24 h of incubation to remove non-adherent cells and provide fresh growth media. After 3 h and 48 h, the coupons were washed with phosphate-buffered saline solution (PBS, Carl Roth, Karlsruhe, Germany), bacterial cells were removed using sonication (3 × 1 min at 35 kHz; Ultrasonic bath, Sonorex RK100H; Bandelin electronic, Berlin, Germany) and drop plated (6 × 5 µL drops) with an electronic multi channel pipette onto TSA (Carl Roth, Germany) and milk agar (Carl Roth, Germany), for *S. capitis* and *M. lacticum*, respectively. Each experiment was repeated in at least triplicates.

### 2.4. Statistical Analysis

For statistical analysis, a multiple range test and Pearson correlation analysis were performed in Statgraphics Centurion XVIII (Statpoint Technologies, Inc., Warrenton, FL, USA). Each experiment was repeated in at least triplicates. All results (*n* ≥ 18) were used for statistical analysis. Statistical significance was considered for *p* ≤ 0.05.

## 3. Results and Discussion

### 3.1. Bacterial Adhesion and Biofilm Formation

*Staphylococcus* spp. and *Microbacterium* spp. are known as good biofilm formers, able to attach well to plastic surfaces and SS, depending on the growth conditions and bacterial strains used [26,27,28,29,30] and are frequently isolated from food contact surfaces in the industry [31,32,33,34]. Furthermore, different *S. capitis* strains showed high resistance against conventional disinfectants, such as chlorhexidine and quaternary ammonium compounds, while *M. lacticum*, adherent to abiotic surfaces, is resistant against cleaning and disinfection and against pasteurization [35,36]. Consequently, these two bacterial strains were selected to investigate the effects of different surface types on biofilm growth.

Adhesion and early-stage biofilm levels between 1.05 ± 0.06 and 2.59 ± 0.11 log CFU cm^−2^ were detected in this study for both bacterial strains. This is similar to levels reported in other studies assessing adhesion and early stage biofilm formation on SS or polymers [37,38]. In general, bacterial attachment to surfaces and subsequently biofilm formation varies with the bacterial strain used and the environmental parameters (i.e., pH, NaCl concentration and temperature) [39]. Similar to other studies published so far [5,40,41,42,43], our data show that differences in bacterial adhesion and early stage biofilm formation are determined by surface type, and bacterial strain tested. Onto sealant surfaces *S. capitis* displayed better biofilm formation capacity compared to *M. lacticum,* which is reflected in the higher number of cells adherent and present in the early-stage biofilm, except for adhesion on S_1_F surface. In the case of SS higher numbers of *S. capitis* cells were detected compared to *M. lacticum* only for adhesion.

**Figure 1:**
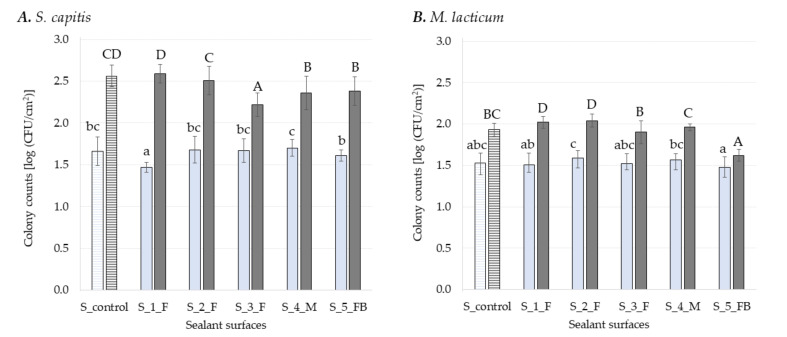
Log CFU cm^−2^ of *S. capitis* (**A**) and *M. lacticum* (**B**) enumerated on different sealant surfaces after 3 h (light blue) and 48 h (dark grey) of incubation. Surfaces with antimicrobial coatings are colored with a solid fill and surfaces without antimicrobials are highlighted with a pattern fill. Different lowercase letters denote significant differences (multiple range test, *p* ≤ 0.05) between the respective samples during adhesion (3 h), while uppercase letters indicate differences in early-stage biofilm formation (48 h). Each experiment was repeated in at least in triplicates.

**Figure 2:**
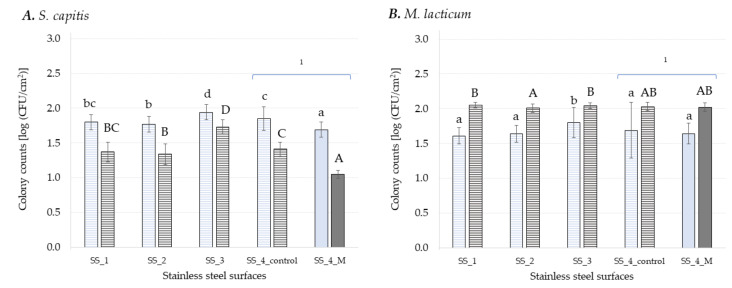
Log CFU cm^−2^ of *S. capitis* (**A**) and *M. lacticum* (**B**) enumerated on different stainless steel surfaces after 3 h (light blue) and 48 h (dark grey) of incubation. Surfaces with antimicrobial coatings are colored with a solid fill and surfaces without antimicrobials are highlighted with a pattern fill. Different lowercase letters denote significant differences (multiple range test, *p* ≤ 0.05) between the respective samples during adhesion (3 h), while uppercase letters indicate differences in early-stage biofilm formation (48 h). Each experiment was repeated in at least triplicates. ^1^ Same SS surface with and without antimicrobial agent.

On average, both bacterial strains attached in slightly lower numbers to sealant surfaces (Figure 1) compared to SS (Figure 2). All sealant surfaces, except for S_control, incorporated either antifungal, antimicrobial or both antifungal and bactericidal agents, while in the case of SS only the surface SS_4_M was coated with antimicrobials (Table 1). This might explain the differences in the number of attached cells detected. Previous studies [42,44,45] show higher attachment and/or biofilm formation onto SS than rubber and/or plasticwhile other studies show the contrary [25,46,47,48,49]. Furthermore, similar biofilm formation on PVC, PE, and SS surfaces was also reported by Zacheus, et al. [50]. Moreover, variation in the assessment method for biofilm formation and types of surfaces tested makes it often difficult to obtain consistent results. Microbial attachment and further biofilm formation are regulated by bacterial cell surface components and by several surface-specific characteristics, such as surface charge and hydrophobicity [6]. For Gram-negative and Gram-positive bacteria, lipopolysaccharides and teichoic acids, respectively, provide charges on the outer surface of bacteria with effect on adhesion and biofilm formation [51]. Most bacteria have a negatively charged and hydrophobic surface at neutral pH and tend to adhere to hydrophobic surfaces rather than hydrophilic ones. However, the degree of cell membrane hydrophobicity depends on the bacterial strain [52,53] and adapts with growth conditions [54]. The hydrophobicity of bacterial strains and surfaces was not characterized within the present study. Even though SS is generally considered hydrophilic, while polymers are known as hydrophobic materials [55], there are studies that found SS type 304 surfaces to be hydrophobic [25,56]. This supports the observations made in the present study. However, the role of hydrophobicity on the bacterial adhesion to a surface is still controversially discussed, as many studies found no relationship between surface properties and biofilm formation [43,45,57,58].

### 3.2. Antimicrobial Sealant Surfaces

Silicones are intensively used in the industry as sealants, being particularly appreciated for their elastic behavior and good resistance to weathering. The name silicone refers to poly-dimethylsiloxanes polymers (PDMS), and by substituting the methyl group along the chain with, e.g., phenyl, vinyl, or trifluoropropyl group, their properties change [59]. Figure 1 presents the number of bacterial cells attached and enumerated in early-stage biofilms subsequently formed onto sealant surfaces. Here, one control surface without antimicrobial agent and five sealants incorporating antimicrobial, antifungal and/or bactericidal agents were investigated. Surprisingly, the number of *S. capitis* cells attached to S_4_M (1.7 ± 0.14 log CFU cm^−2^), a sealant material incorporating an antimicrobial agent, displayed the highest number detected among all sealant surfaces, being significantly higher (*p* ≤ 0.05) compared to S_5_FB (1.61 ± 0.07 log CFU cm^−2^) and S_1_F (1.47 ± 0.17 log CFU cm^−2^), with 5% and 14% higher CFU cm^−2^ counts, respectively. However this was not significantly different (*p* > 0.05) when compared to the control (S_control, the sealant surface of MS polymer-basis without any antimicrobial agent). For *M. lacticum*, the highest number of adherent cells was found onto S_2_F (1.59 ± 0.09 log CFU cm^−2^), while the lowest number was found onto S_5_FB (1.48 ± 0.12 log CFU cm^−2^). In this case the presence of both fungicidal and bactericidal active ingredients reduced the number of bacterial cells attached to S_5 _FB by 7% when compared to S_2_F, while for S_4_M only 1% bacterial cell reduction was seen. Furthermore, none of the tested surfaces incorporationg antimicrobial and/or antifungal compounds was significantly different to the control surface (S_control).

Counts of bacterial cells in early-stage biofilms (48 h) formed on sealants ranged between 1.62 ± 0.07 log CFU cm^−2^ for S_5_FB and 2.04 ± 0.08 log CFU cm^−2^ for S_2_F in case of *M. lacticum*. Incorporation of antibacterial agents into S_5_FB and S_4_M, reduced, therefore, the early-stage biofilm formation of *M. lacticum* by 21% and 4% respectively as compared to S_2_F. However, compared to the control surface (S_control) a significant reduction of 16% (*p* ≤ 0.05) was seen in the number of cells in the early-stage biofilm formed onto S_5_FB, but not onto S_4_M surface. The S_5_FB surface is the only one incorporating both fungicidal and antibacterial agents, most likely resulting in synergic effects in reducing biofilm formation of *M. lacticum*. Previous reports described synergic interactions between antibacterial and antifungal compounds [60,61]. However, such effects of S_5_FB on *S. capitis* biofilm growth was not observed. Therefore, it is assumed that the effects are bacterial strains specific. In future research, it would be also of interest to assess fungi or fungal spores to obtain holistic information on the effectiveness of the surface agents. Early-stage biofilms formed onto S_1_F and S_2_F, both incorporationg an antifungal agent were including significantly (*p* ≤ 0.05) higher numbers of cells compared to the control (S_control). For *S. capitis*, the highest mean value in the early-stage biofilm (48 h) was observed onto S_1_F (2.59 ± 0.11 log CFU cm^−2^) and the lowest mean value onto S_3_F (2.22 ± 0.14 log CFU cm^−2^) (Figure 1).

Incorporation of an antibacterial agent into surfaces S_4_M and S_5_FB reduced the number of bacterial cells in the biofilm developed on these two surfaces by 7% compared to the control surface (S_control). Furthermore, a significant reduction (*p* ≤ 0.05), of 13%, could be seen also in the number of cells in the early-stage biofilm formed onto S_3_F compared to S_control. Previous studies already showed that different combinations of polymers with antimicrobial agents efficiently reduce biofilm formation [5,62,63]. For example, immobilization of polymyxins on PDMS was able to prevent the adhesion of *Pseudomonas aeruginosa* and inactivated a significant fraction of the adherent cells [62]. Moreover, it was found that surface coating with 2-methacryloyloxyethyl phosphorylcholine co-polymer significantly reduces retention of human pathogenic microorganisms [63]. For antimicrobial polymer surfaces, polymer charge and hydrophobicity have been identified as the leading parameters that affect antimicrobial activity. These surfaces bear cationic charges and kill or deactivate bacteria by interaction with negatively charged parts of their cell envelope [64]. According to Hyde, et al. [65], biofilm adherence to polymer-based surfaces is a function of both surface finish and surface chemistry. Assessment of surface-specific properties was not performed in this study. However, the substratum surface properties, like hydrophobicity or roughness, are generally impacting the late-stage rather than the early-stage biofilm formation [3], and surface roughness of material may hinder effective cleaning and sanitizing and not biofilm formation [66,67].

### 3.3. Antimicrobial Stainless Steel Surfaces

Stainless steel (SS) is a common food contact material, regularly used for process equipment because of its corrosion resistance, cleanability and high mechanical strength. Here, four SS surfaces with varying thickness in the coating layer (SS_1, SS_2 and SS_4; 25, 50 and 150 µm) and additional alloy (SS_3; Standard V2A) were tested. Additionally to the SS with 150 µm thick coating (SS_4_control; 150 µm) a surface with the same coating and additional antimicrobial agents was tested (SS_4_M).

The number of cells attached to the SS surfaces and the early stage biofilm formed onto these surfaces is depicted in Figure 2. The lowest adhesion rate of *S. capitis* on SS surfaces was detected for SS_4_M (1.69 ± 0.11 log CFU cm^−2^), which was significantly reduced (*p* ≤ 0.05) compared to the control surface (SS_4_control) and also compared to the other SS surfaces without antimicrobials with a percentage of 5% to 13%. The highest adhesion rate was seen for SS_3 (1.94 ± 0.11 log CFU cm^−2^). Biofilm formation of *S. capitis* showed a similar trend with significantly (*p* ≤ 0.05) lowest cell density on SS_4_M (1.05 ± 0.06 log CFU cm^−2^) and with significantly (*p* ≤ 0.05) highest cell population on SS_3 (1.73 ± 0.1 log CFU cm^−2^). Furthermore, the antimicrobial agent used to coat the SS_4_M reduced the number of bacterial cells in the early-stage biofilm by 26% in comparison to SS_4_control (=control surface with the same base but without antibacterial agent). Furthermore, the size of coating applied on SS_1 (25 µm) and SS_2 (50 µm) reduced significantly (*p* ≤ 0.05) the number of attached cell onto these surfaces when compared to SS_4_control (140 µm), but significantly increased the number of cells in the early-stage biofilm formed onto these surfaces. Surprisingly, the mean value of *S. capitis* cells detected in early stage biofilm (after 48 h) was significantly (*p* ≤ 0.05) lower compared to attachment rate (after 3 h) on SS surfaces (Figure 2A). This can be related to the static biofilm system used, where the availability of nutrients might be a limiting factor during biofilm growth [3], as *S. capitis* has a faster growth rate when compared to *M. lacticum* [25]. It is considered that under static conditions, adherent cells may be present in high numbers but do not always increase over the incubation time as a consequence of the cell division process and/or redistribution of adherent cells forming the biofilm [68]. Therefore, high levels of initial adherence do not necessarily lead to a thicker and stronger biofilm matrix [69]. This was also seen in the present study for sealants (Figure 1). The lowest adhesion rate of *S. capitis* on S_1_F (1.47 ± 0.17 log CFU cm^−2^) led to the highest cell population in early stage biofilm (2.59 ± 0.11 log CFU cm^−2^). Furthermore, some of the cells within a biofilm might be in the viable but non-culturable (VBNC) state and hence, cannot be detected by plating [70].

Despite some limitations, the plate count method used in this study is still the gold standard for microbiological measurements, especially for industrial uses, mainly because it is cost-efficient, easy to apply, and allows adaptation to a variety of conditions showing reliable results [3]. Overall, bacterial adhesion on sealants (Figure 1) was lower compared to adhesion on SS (Figure 2), for surfaces with and without antimicrobials, while antimicrobial coatings on SS surfaces played a role in reducing early-stage biofilm formation of *S. capitis*. Their different effects on the two Gram-positive bacteria are most likely related to the different mechanisms of action of incorporated antimicrobials vs. antimicrobial coatings [71].

No significant difference (*p* > 0.05) in the mean value of attached cells was found for *M. lacticum* among the SS surfaces, except for SS_3. *M. lacticum* cells in early-stage biofilm formed on SS_2 (2.01 ± 0.06 log CFU cm^−2^) were by 2% significantly lower (*p* ≤ 0.05) when compared to SS_1 (2.05 ± 0.04 log CFU cm^−2^) and SS_3 (2.04 ± 0.04 log CFU cm^−2^) (Figure 2). Because of cracks and crevices on its surface, SS is prone to bacterial attachment and biofilm formation. Several publications reported that antibiofilm coatings of SS are successful in preventing adhesion and biofilm formation of different bacterial strains [22,23,72,73]. In this study, the addition of an antimicrobial agent to the PVC foil (SS_4_M) significantly reduced (*p* ≤ 0.05) the number of *S. capitis* cells attached as well as the number of cells in early-stage biofilm compared to the control (SS_4_control, coated only with PVC foil), by 8% and by 25%, respectively (Figure 2). For *M. lacticum*, no significant (*p* > 0.05) difference could be detected, neither for adhesion nor early stage biofilm formation by the addition of antimicrobial agents in the PVC coating. Furthermore, maximum cell counts were recorded for both adhesion and biofilm formation on SS_3 (the standard SS Niro Duplo V2A) independent of the bacterial strain used. Flint, et al. [74] reported that the natural oxide coat on SS enhances adhesion of thermo-resistant streptococci, and this can be reduced by removing the oxide layer. Besides the antimicrobial agent and surface characteristics, growth conditions impact on the antimicrobial effect [75]. Furthermore, the thickness of coating layer and molecular weight of the coating polymer can cause differences in surface coverage or modulus and hence influences bacterial adhesion and biofilm formation onto surface [64]. In this study, weak positive correlations (0.30) between the thickness of the coating layer for SS_1 (25 µm), SS_2 (50 µm), and SS_4_control (150 µm) and rate of adhesion and cell population in early-stage biofilms, respectively, were seen only for *S. capitis.* Al-Ahmad, et al. [76] noted that antimicrobial activity of various antimicrobial polymer-coated silicones was about the same but, inactivation kinetics varied significantly with layer thickness. The 50 nm and 150 nm thick networks were able to kill 17% and 63% of the adherent *S. aureus* cells in 3 min, respectively. The difference could be related to variations in bacteria-surface contact area or to stiffness of substrate, on which the networks were built.

Moreover, it should be noted that antimicrobial surface agents can inhibit biofilm formation for example via quorum sensing [77] or cyclic di-GMP (c-di-GMP) signaling interference, of which the latter is an intracellular signaling molecule of bacteria [78]. Quorum sensing (QS) is an intra- and intercellular mechanism, which facilitates the communication between cells and the interaction between the environment and cells [79]. The QS mechanism differs between bacteria species but can be organized in three categories: 1) the autoinducer-2 (AI-2) system, observed in interspecies interactions of both Gram-positive and Gram-negative bacteria, 2) the acyl-homoserine lactone (AHL) or autoinducer-I (AI-I) system found in Gram-negative bacteria, and 3) the peptide-mediated QS system observed in Gram-positive bacteria [80]. Since these mechanisms were not investigated in the present study, they are not discussed in more detail at this point, but should be addressed in future research.

## 4. Conclusions

In the present study, surface conditioning by incorporating antimicrobial agents was more efficient in reducing bacterial adhesion compared to active antimicrobial coatings. In contrast, active antimicrobial coatings played a role in reducing early-stage biofilm formation. Based on the present findings, antimicrobial surfaces cannot replace cleaning and disinfection strategies, but can be used as an additional tool to reduce bacterial adhesion and biofilm formation. Therefore, food producers and equipment manufacturers should carefully select antimicrobial surfaces for their intended use.

However, this study is also limited to the effects of antimicrobial agents on two Gram-positive biofilm formers. Hence, future studies should consider mixed biofilms consisting of Gram-positive and Gram-negative bacteria as well as fungi, especially for antifungal agents, to validate the present results. Additionally, an in-depth analysis of the antibacterial effect on bacterial attachment and biofilm growth, including microscopic evaluation and detailed characterization of the surface properties (e.g., hydrophobicity) should be considered.

## Figures and Tables

**Table 1:** Detailed information about the tested sealant and stainless steel surfaces with or without antimicrobial, -fungal or/and bactericidal components according to the manufacturer’s data; The different antimicrobial surfaces are marked as followed: F—antifungal, M—antimicrobial, FB—fungicidal and bactericidal. ^1^ Same surface type with and without antimicrobial coating.

Surface Type	Commercial Name	Short Name	Composition Basis	Antimicrobial Substance(s)
Sealant (S)	EVT Hybrid glue 007	S_control	1K MS Polymer	-
	EVT Joint HPA	S_1_F	One component elastic silicone—oxime base	Incorporated antifungal agent
	EVT Clean Room Sanitary HPCR	S_2_F	One component elastic silicone—oxime base	Incorporated antifungal agent
	Sanitary HPS silver	S_3_F	One component elastic silicone—acetate base	Incorporated antifungal agent
	Novasil M-SP7389	S_4_M	One component adhesive silicone—Hybrid polymer STP base	Incorporated antimicrobial agent
	Sanitary 450 white	S_5_FB	Acid-curing acetoxy system, acetate base	Incorporated fungicidal andbactericidal agents
Stainless steel (SS)	SS with polyester foil of 25 µm thickness	SS_1	Polyester polyethylene	-
	SS with polyurethan-polyamide (PP) foil of 50 µm	SS_2	Polyurethan-polyamid	-
	SS Niro Duplo V2A	SS_3	Stainless steel standard	-
	SS polyvinyl chloride (PVC) foil 150 µm^1^	SS_4_control	Hard PVC folie coating	-
	SS with PVC foil of 150 µm thickness—antibac ^1^	SS_4_M	Hard PVC folie coating	antimicrobial coating
